# The added value of One Health surveillance: data from questing ticks can provide an early signal for anaplasmosis outbreaks in animals and humans

**DOI:** 10.17269/s41997-022-00723-8

**Published:** 2022-12-05

**Authors:** Jérôme Pelletier, Camille Guillot, Jean-Philippe Rocheleau, Catherine Bouchard, Geneviève Baron, Christian Bédard, Antonia Dibernardo, L. Robbin Lindsay, Patrick A. Leighton, Cécile Aenishaenslin

**Affiliations:** 1 Département de pathologie et microbiologie, Faculté de médecine vétérinaire, Université de Montréal, Saint-Hyacinthe, Québec Canada; 2 Groupe de recherche en épidémiologie des zoonoses et santé publique, Faculté de médecine vétérinaire, Université de Montréal, Saint-Hyacinthe, Québec Canada; 3 Centre de recherche en santé publique de l’Université de Montréal et du CIUSSS du Centre-Sud-de-l’Île-de-Montréal, Université de Montréal, Montréal, Québec Canada; 4 Département de santé animale, CÉGEP de Saint-Hyacinthe, Saint-Hyacinthe, Québec Canada; 5 Public Health Risk Sciences Division, National Microbiology Laboratory, Public Health Agency of Canada, Saint-Hyacinthe, Québec Canada; 6 Direction de la santé publique du CIUSS de l’Estrie, Sherbrooke, Québec Canada; 7 Département des sciences de la santé communautaire, Faculté de médecine et des sciences de la santé, Université de Sherbrooke, Sherbrooke, Québec Canada; 8 Centre de diagnostic vétérinaire de l’Université de Montréal, Saint-Hyacinthe, Québec Canada; 9 One Health Division, National Microbiology Laboratory, Public Health Agency of Canada, Winnipeg, Manitoba Canada

**Keywords:** *Ixodes scapularis*, Granulocytic anaplasmosis, Canada, *Anaplasma phagocytophilum*, *Peromycus* mice, One Health, *Ixodes scapularis*, Anaplasmose granulocytaire, Canada, *Anaplasma phagocytophilum*, *Peromycus* spp., Une seule santé

## Abstract

**Objective:**

In 2021, a first outbreak of anaplasmosis occurred in animals and humans in southern Québec, with 64% of confirmed human cases located in Bromont municipality. *Ixodes scapularis* ticks and *Peromyscus* mouse ear biopsies collected in Bromont from 2019 to 2021 were analyzed for *Anaplasma phagocytophilum* (Ap) with the objective of determining whether an early environmental signal could have been detected before the outbreak.

**Methods:**

Samples were collected for a concurrent study aiming to reduce Lyme disease risk. Between 2019 and 2021, up to 14 experimental sites were sampled for ticks and capture of small mammals took place on three sites in 2021. Samples were screened for Ap using multiplex real-time PCR, and genetic strains were identified using a single-nucleotide polymorphism assay.

**Results:**

Analyses showed an increase of 5.7% in Ap prevalence in ticks (CI95: 1.5–9.9) between 2019 and 2020, i.e., one year before the outbreak. A majority of Ap-positive ticks were infected with the zoonotic strain (68.8%; CI95: 50.0–83.9) during the study period. In 2021, 2 of 59 captured *Peromycus* mice were positive for Ap, for a prevalence of 3.4% (CI95: 0.4–11.7).

**Conclusion:**

We conclude that data collected in Bromont could have provided an early signal for an anaplasmosis risk increasing in the targeted region. This is a reminder that integrated surveillance of tick-borne diseases through structured One Health programs, i.e. systematically integrating data from humans, animals and the environment, can provide useful and timely information for better preparedness and response in public health.

**Supplementary Information:**

The online version contains supplementary material available at 10.17269/s41997-022-00723-8.

## Résumé

**Objectif:**

En 2021, suivant une éclosion d’anaplasmoses chez les animaux et les humains dans le sud du Québec, des tiques de l’espèce *Ixodes scapularis* et des biopsies de souris *Peromyscus* spp. échantillonées à Bromont, la municipalité où 64 % des cas humains confirmés était localisé, ont été testées pour *Anaplasma phagocytophilum* (Ap) avec pour objectif de déterminer si un signal environnemental précoce d’augmentation du risque aurait pu être détecté avant l’éclosion.

**Méthode:**

L’échantillonnage a été réalisé dans le cadre d’une étude visant à réduire le risque de maladie de Lyme. De 2019 à 2021, 14 sites expérimentaux ont été échantillonnés pour les tiques. En 2021, trois sites ont été sélectionnés pour la capture des micromammifères. Les échantillons ont été testés pour la présence d’Ap à l’aide d’un PCR multiplex en temps réelle et les lignées génétiques ont été identifiées grâce à un test de polymorphisme mononucléotidique.

**Résultats:**

Les analyses ont montré une augmentation de 5,7 % (IC95% : 1,5–9,9) de la prévalence de Ap entre 2019 et 2020, c’est-à-dire un an avant l’éclosion. Cette augmentation est associée à la présence d’une majorité d’Ap de la lignée zoonotique (68,8 %; IC95% : 50,0–83,9) sur l’ensemble de la période étudiée. En 2021, deux *Peromycus* spp. capturées sur 59 étaient positives pour Ap pour une prévalence de 3,4 % (IC95% : 0,4–11,7).

**Conclusion:**

Les données environnementales échantillonnées à Bromont auraient pu fournir un signal précoce de l’augmentation du risque d’anaplasmose dans la région. C’est un rappel que la surveillance intégrée des maladies transmises par les tiques inspirée de l’approche Une seule santé, intégrant systématiquement des données humaines, animales et environnementales, peut fournir des informations utiles et opportunes aux autorités de santé publique.

## Introduction

Rising temperature due to climate change is a major factor explaining the northward expansion of *Ixodes scapularis* ticks in Canada (Bouchard et al., 2019). Currently, established populations of *I*. *scapularis* can be found in several Canadian provinces and are driving the emergence of tick-borne pathogens (TBP) that are of concern to both animal and human populations (Bouchard et al., 2019). In the past 20 years, the bacteria *Borrelia burgdorferi*, the etiological agent of Lyme disease (LD), followed the geographic range expansion of the *I*. *scapularis* population in Canada and became a tick-borne disease (TBD) of major concern for public health with an annual number of cases that increased from 266 in 2011 to 2851 in 2021 (Government of Canada, 2021). Concurrently, the emergence of other pathogens transmitted by *I*. *scapularis* ticks such as *Anaplasma phagocytophilum* (Ap) or *Babesia microti* in southern Canada remains a growing public health concern due to their associated morbidity and mortality (Bouchard et al., 2019). For example, human granulocytic anaplasmosis (HGA), caused by Ap, is characterized by unspecific symptoms such as headache, generalized myalgias, or rigours, and is life threatening in 3% of cases and fatal in 1% (Bakken & Dumler, 2015).

Since the recorded presence of *I*. *scapularis*, different genetic strains of Ap have been circulating in Canada, including a zoonotic strain qualified as human active (Ap-ha) and a non-zoonotic strain classified as Ap-variant 1 (Massung et al., 2002). The first is maintained in the environment by a variety of vertebrate species, including small mammals such as *Peromyscus* mice, shrews, chipmunks, or squirrels, and causes a clinical disease in humans, dogs, and horses (Keesing et al., 2014). Ap-variant 1 is maintained in the environment by white-tailed deer and has not been shown to be associated with clinical disease in humans, dogs, or horses as it has a tropism for ruminant species (Morissette et al., 2009; Trost et al., 2018). Krakowetz et al. (2014) tested for Ap strains in 145 ticks collected between 2007 and 2010 across Canada. The study found the proportion of Ap-ha to be 36.8% while 63.3% were Ap-variant 1 (Krakowetz et al., 2014).

Before the first reported cases of HGA in Nova Scotia and Ontario, respectively, in 2017 and 2018, HGA in Canada had only been reported in western Canadian provinces (Edginton et al., 2018; Manitoba Health, 2019; Nova Scotia Zoonotic Technical Working Group, 2021; Parkins et al., 2009; Stokes et al., 2020). HGA is a reportable disease in only two Canadian provinces. In Manitoba, where it is reportable since 2015, 59 probable (*n* = 20) and confirmed (*n* = 39) cases have been reported so far (Manitoba Health, 2019). In Québec, HGA became a notifiable disease in 2019 and, between that year and 2020, only 5 cases had been reported, of which a single case was confirmed as locally acquired (Ouhoummane et al., 2020, 2021). Nonetheless, in this province, serological investigations have detected evidence of exposure to Ap in dogs as early as 2008 and in humans since 2014 (Villeneuve et al., 2011). This is consistent with the detection of Ap in tick populations in Québec since 2007 (Krakowetz et al., 2014). Although past data suggest that Ap-variant 1 has been the main strain found in Québec (80.9%, 38/47), identification of the Ap strain is not routinely performed in ticks from TBD surveillance programs; therefore, their relative distribution and proportion have yet to be established (Krakowetz et al., 2014).

In 2021, an unusual outbreak of locally acquired cases of animal granulocytic anaplasmosis (AGA) and HGA was reported in the region of Estrie, Québec. Initially, AGA cases (one dog and two horses) from this region were diagnosed between April and June at the Centre de diagnostic vétérinaire de l’Université de Montréal. The total number of animal cases remains unknown because AGA is not a reportable disease. Between May and November, 25 confirmed HGA cases, including 11 hospitalizations, were confirmed by Estrie Public Health Authority (Campeau et al., 2022). All AGA or HGA cases were residents of Estrie or reputed to have visited the region delimited by two local health service units named La Pommeraie and Haute-Yamaska. The municipality of Bromont was mostly affected, with 64% of HGA confirmed cases (Campeau et al., 2022). Even though this region is known to have high LD incidence in humans, the sudden outbreak of human GA cases generated concerns in the population.

As part of a concurrent research project that aims to evaluate a preventive intervention for LD in Bromont, our team collected questing nymphal and adult *I*. *scapularis* ticks from 2019 to 2021 (*n* = 1934), and ear biopsies from captured *Peromyscus* spp. mice in 2021 (*n* = 59). Following the first reports of AGA cases in spring 2021, we tested a subsample of ticks (*n* = 870, 45%) for Ap, with the objective of detecting changes in the prevalence of ticks infected with Ap and to identify Ap strains. All 59 *Peromyscus* spp. mice ear biopsies were also tested for Ap to determine whether the pathogen could be detected in this wildlife reservoir. The overall goal was to determine whether an early signal of increasing Ap risk could have been detected before the occurrence of the AGA and HGA outbreak. This article presents the main findings of these analyses and builds on this unexpected situation to underline the added value of One Health surveillance for the early detection of emerging TBP.

## Methods

### Study site

**Fig. 1:**
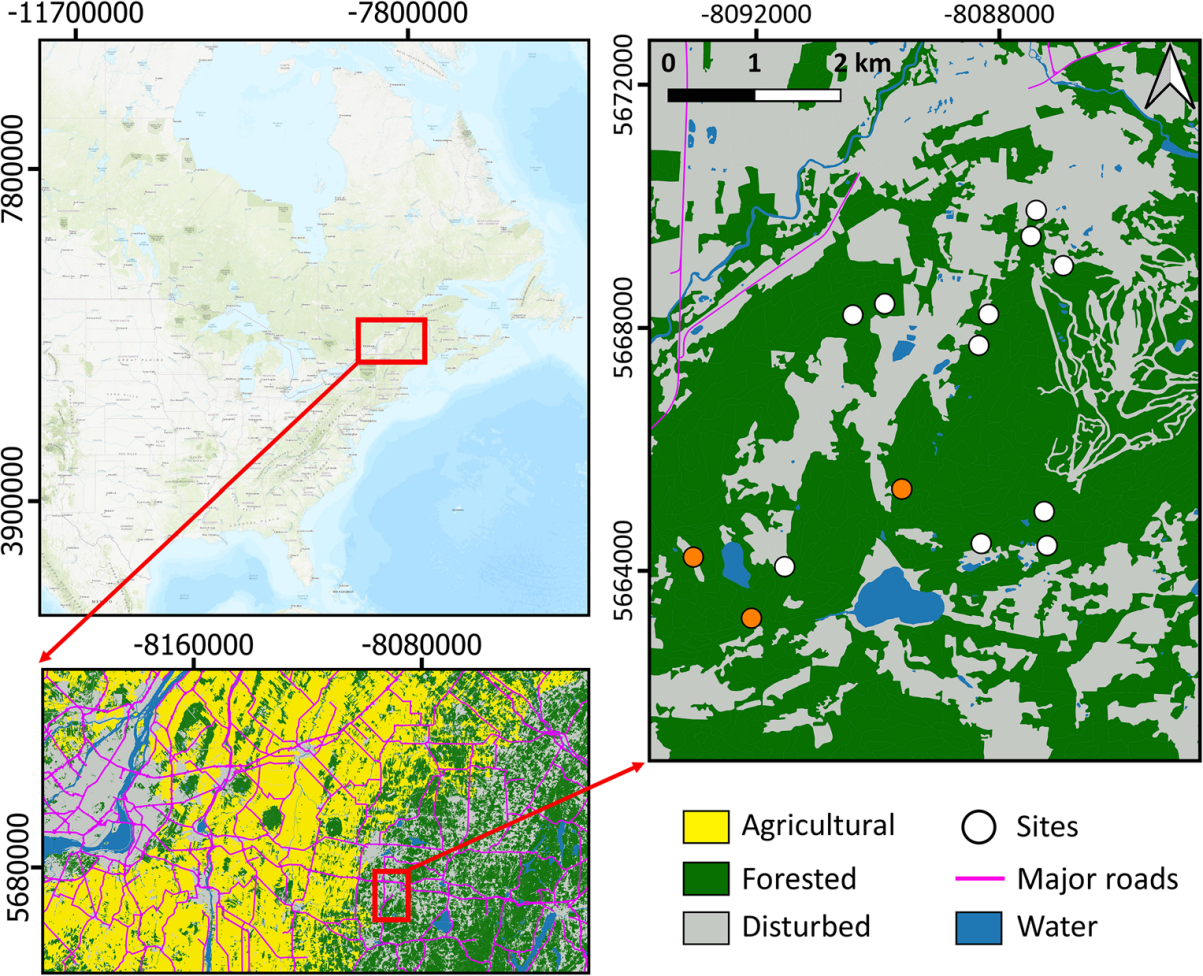
Location of the experimental area in southern Québec, Canada, and of experimental sites within the experimental area. Dots filled with orange represent the experimental sites where small mammals were captured in 2021

The municipality of Bromont is a small suburban community of 11,357 inhabitants with a population density of 99.6 inhabitants by square kilometre located in southern Québec, 115 km north of the Canada-United States border (Fig. 1) (Statistics Canada, 2022). The majority of neighbourhoods are composed of areas with low household densities within large patches of mixed deciduous forests that provide thick dead leaf litters favouring *I*. *scapularis* tick survival. Bromont is part of the broader region of La Pommeraie which is a known endemic area for LD with an incidence of 357.3 per 100,000 people in 2021 (Baron et al., 2022).

### Sampling

Ticks and mice ear biopsies were collected as part of another study aiming to evaluate the effect of administrating acaricidal baits to small mammals on tick prevalence of infection with *Borrelia burgdorferi* (for details, see the Supplementary file). Up to 14 experimental sites were included in the main study from 2019 to 2021 (Fig. 1). Sites were included in the study because they present ecological characteristics compatible with high Lyme disease risk, i.e., maple forests with thick leaf litter, and identified as such by community stakeholders. Ticks were sampled systematically using a 1 m^2^ piece of white flannel fabric dragged horizontally on the forest ground (Rulison et al., 2013). Questing nymph and adult ticks were counted and collected every 25 m. Specimens were stored in 70% ethanol to allow for later species identification and subsequent pathogen testing. In 2021, capture of small mammals took place on three selected sites (Fig. 1). In total, 100 Sherman traps (H.B. Sherman Traps, Tallahassee, FL, USA) were deployed during two capture periods for each of the three sites: once in July and once in August. An ear biopsy was taken when a *Peromyscus* mouse was captured for the first time and conserved in 70% ethanol until pathogen testing.

### Ap testing

Samples were conserved at room temperature for a maximum of 2 years. If a site had less than 30 ticks in a year, all samples were tested. If more than 30 ticks had been collected, a subsample of 30 to 40 ticks was randomly selected. Ticks from 2019 and 2020 were cut in half; one half was saved for the main study and the other was tested for the current investigation. Whole ticks were tested for the year 2021. In brief, DNA was extracted with DNeasy 96 tissue kits (Qiagen, Mississauga, ON, Canada) according to the manufacturer protocol. Samples were screened for Ap using multiplex real-time PCR (Courtney et al., 2004). Ap strains were identified with a single-nucleotide polymorphism assay targeting the 16S rRNA gene developed by Krakowetz et al. (2014).

### Data analysis

Prevalence of infection with Ap in *I*. *scapularis* ticks and *Peromyscus* spp. mice was computed by year. First, 95% confidence intervals (CI95) of the prevalence were computed with binomial exact test. Statistical significance in prevalence variation was tested with Pearson chi squared when exact CI95 were not overlapping to limit multiple testing. The distribution of ticks infected with Ap and Ap-ha strain among study sites was described. Statistical analyses were performed with R software version 4.1.1 (R Core Team, 2017).

## Results

### Tick samples

**Fig. 2:**
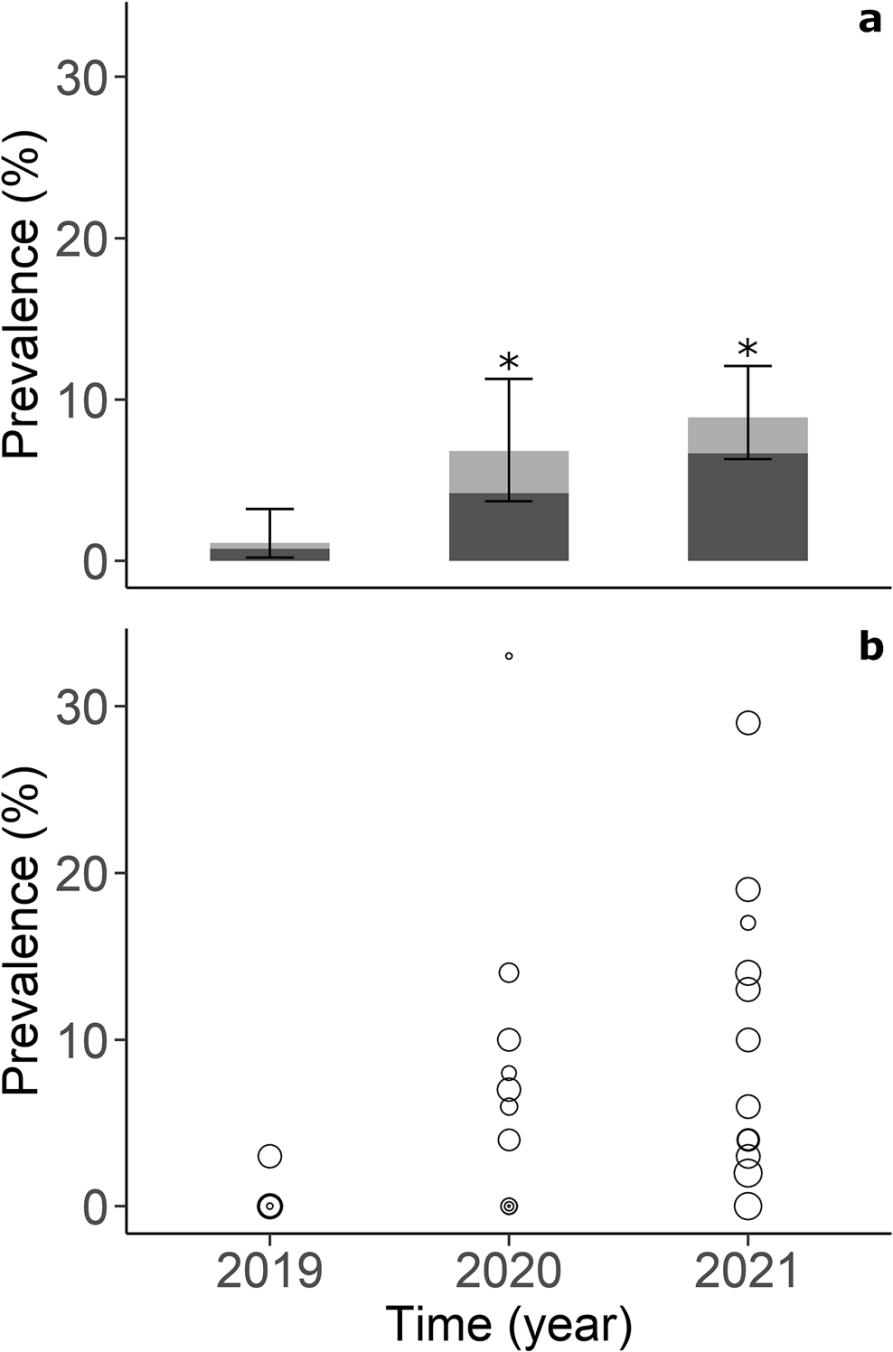
Prevalence of *A*. *phagocytophilum* (**a**) and the distribution of experimental plots’ average prevalence per year (**b**). In panel **a**, the proportion of light versus dark gray represents, respectively, the proportion of Ap-variant 1 and Ap-ha among the samples with conclusive tests. In panel **b**, each dot represents an experimental site and the dot size is proportional to the number of ticks tested. * indicates a significant difference with year 2019 (Pearson chi^2^, *p* < 0.05)

**Fig. 3:**
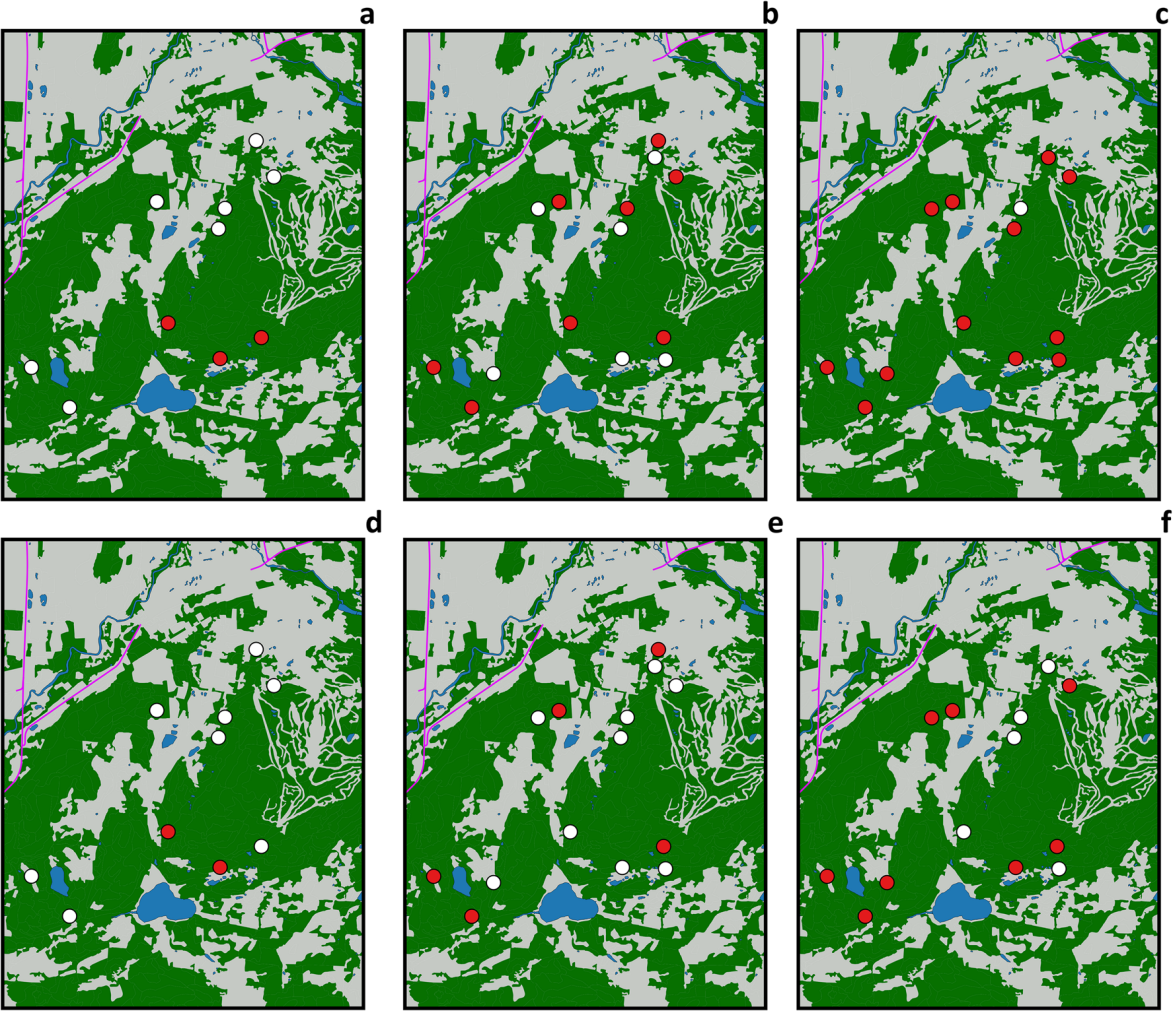
Distribution of *A*. *phagocytophilum* and *A*. *phagocytophilum* human active strain in experimental sites. In the upper panels, the distribution of Ap in 2019 (**a**), 2020 (**b**), and 2021 (**c**). In the lower panels, the distribution of Ap-ha in 2019 (**d**), 2020 (**e**), and 2021 (**f**). Dots filled with red indicate experimental sites with at least one positive tick for Ap or Ap-ha

Tests were able to conclusively identify the specific Ap strain for 32 out of 52 specimens positive for Ap. Both Ap-ha and Ap-variant 1 were detected in adult and nymph ticks collected each year; overall, the proportion of Ap-ha was found to be 68.8% (*n* = 22) among these tick specimens (Table 1). Over the 3 years of the study, Ap prevalence was at its lowest in 2019 (1.1%; CI95: 0.2–3.2) but increased significantly in 2020 (6.8%; CI95: 3.5–11.3) and in 2021 (9.5%; CI95: 6.2–13.9). These changes correspond to an increase of 5.7% in 2020 (CI95: 1.9–9.4) and 8.4% in 2021 (CI95: 4.5–11.2) when compared with 2019 (Fig. 2a). Three sites out of 10 had at least 1 positive tick for Ap in 2019, 8 out of 14 in 2020, and 12 out of 13 in 2021. Two sites out of 10 had at least 1 Ap-ha-infected tick in 2019, 4 out of 14 in 2020, and 8 out of 13 in 2021 (Fig. 3). The median (range) of Ap prevalence by sites was 0% (0–3%), 5% (0–33%), and 6% (0–29%) in 2019, 2020, and 2021, respectively (Fig. 2b).

**Table 1:** The number of *Anaplasma phagocytophilum*–positive ticks and the proportion of human active strain by year

Year	Ap^a^	Ap-ha^a^	Ap-ha/Ap (%) [CI95]	No. tested [%]	No. collected
2019	3	2	66.7 [9.4–99.2]	274 [31.2]	877
2020	13	8	61.5 [31.6–86.1]	192 [89.3]	215
2021	36 [16]^b^	12	75 [47.6–92.7]	404 [48.0]	842
Total	52 [32]^b^	22	68.8 [50.0–83.9]	870 [45.0]	1934

^a^Number of Ap- or Ap-ha-positive ticks

^b^Numbers in brackets relate to the number of Ap-positive samples for which the specific strain was successfully identified. In 2021, 20 tests out of 36 were inconclusive due to too small amounts of DNA and were excluded from proportion calculations

### Biopsy samples

From the ear biopsies collected in 2021, 2 out of 59 *Peromyscus* mouse ear biopsies tested positive for Ap, for an Ap infection prevalence of 3.4% (CI95: 0.4–11.7). No Ap-positive samples were successfully identified for strains.

## Discussion

In this article, we describe the changes over time in the prevalence of Ap infection in questing ticks in a region where an outbreak of AGA and HGA cases (Campeau et al., 2022) was recently identified. The findings show a significant increase in Ap prevalence between 2019 and 2021. Strain identification revealed that Ap-ha was involved in a majority of Ap-positive ticks (68.8%). Interestingly, this increase was already noted in 2020, i.e., one year before the unusual outbreak of AGA and HGA. We believe that this finding underlines the relevance of implementing and maintaining One Health surveillance, i.e., surveillance in which collaborative efforts exist between at least two sectors (among human health, animal health, plant health, food safety, wildlife and environmental health) to produce and disseminate information with the purpose of improving human, animal, or environmental health (Bordier et al., 2020). Specifically, in southern Québec, i.e., where Ap is a new emerging TBD, we demonstrate that the routine collection and analysis of data on Ap circulation in ticks in the environment could have provided an early signal of increasing risk of AGA and HGA.

Such a surveillance system exists for monitoring LD emergence in southern Québec. The program integrates data collected actively through questing ticks sampled in the environment, passive surveillance data from ticks found on humans or domestic animals, and reported human cases of LD (Adam-Poupart et al., 2016). This system has the main objective of following the emergence of LD, and thus, surveillance activities are centered in areas where it is not endemic or has not yet been identified (Bouchard et al., 2019). For instance, passive tick surveillance was interrupted in regions where LD pathogens are known to be endemic and submissions originating from domestic animals are no longer routinely analyzed. This impairs the possibility of detecting other emerging TBP in these regions.

In the last 10 years, incidence rates of HGA rapidly increased in New York and New England states (Elias et al., 2020; New Hampshire Division of Public Health Services, 2018; Russell et al., 2021; Vermont Department of Health, 2022). Based on the history of *B*. *burgdorferi* emergence, it is plausible that the Ap epidemiological situation in New York and New England is a good indicator of what will happen in southern Québec, as they share borders and similar ecosystems. We argue that there are several benefits of maintaining or increasing surveillance resources in southern Québec regions where LD is already endemic, as it may allow early detection of new TBD that might eventually expand northward.

Another TBD surveillance program, the Canadian Lyme Sentinel Network (CaLSeN), has been in place since 2019 (Guillot et al., 2020). It uses a sentinel approach to provide comparative measures of LD risk by active sampling of questing ticks. Between 2019 and 2021, Québec had four sentinel regions, two of which are located in Estrie (Guillot et al., 2020). In this period, CaLSeN surveillance included 10 sites sampled twice each summer in Estrie; 1 or 2 of them were located in Bromont. Of ticks collected from those sites: 2.3% (1/43), 0% (0/11), and 4.2% (2/71) were positive for Ap in 2019, 2020, and 2021, respectively (unpublished data). CaLSeN did not detect an increase in Ap prevalence in 2020, but data still suggest a slight increase in Ap prevalence in 2021 in this region even if interpretation remains limited by the low number of ticks available for testing, especially in 2020. The lack of detection of Ap-prevalence increase in 2020 could be explained by two factors: (i) lower sampling efforts and (ii) the location of sampling sites that are not solely centered within an area with a high TBD risk. In comparison, CaLSeN surveillance in southern Québec totalled 10,000 m^2^ of sampled surfaces per year between 2019 and 2021, while the study in Bromont had between 47,000 and 93,000 m^2^ on sites specifically designed to be located in an area with a high TBD risk. It is important to note that CaLSeN sampling had been greatly reduced in 2020 due to constraints imposed by the pandemic that happened concurrently with an atypical low density of questing ticks. However, the capacity of CaLSeN to detect the circulation of emerging TBP could be increased by strengthening the sampling efforts and by re-orienting the location of sentinel sites toward known endemic regions for LD in the future.

This article is an important reminder that allocating sufficient resources to the surveillance of questing ticks and to tick testing in a timely manner is critical to inform early and effective public health responses. As a matter of fact, in the case of Ap, actual surveillance programs do not perform strain identification in a timely manner, but this information is a necessity to properly characterize AGA and HGA hazard in the environment (Krakowetz et al., 2014).

More broadly, the recent outbreak and our results raise the question of granulocytic anaplasmosis emergence in southern Canada. The transmission cycle of the zoonotic strain of Ap is driven by *I*. *scapularis* ticks and a variety of vertebrate reservoirs, such as *Peromyscus* mice (Keesing et al., 2014), but factors causing its emergence in this region remain unknown. Actually, the emerging situation of anaplasmosis in southern Québec and Canada might represent an opportunity to investigate Ap strain ecology and the specific determinants of their distribution in the environment. Despite the fact that our analyses described the presence of the pathogens in one of their reservoirs and identified 3.4% of Ap-positive *Peromyscus* mice, the study was not designed to investigate the ecological determinants of this pathogen. Results presented in this article remain limited by their opportunistic nature. The main study aims to investigate the effect of treating with acaricides small mammals on Lyme disease risk. The effect of this intervention on the prevalence of Ap in tick populations is unknown.

Information reported in this article remains crucial by highlighting changes in the epidemiological situation of Ap and Ap-ha in southern Québec. The increase in Ap prevalence in questing ticks is likely to be one of the drivers of the first outbreak of AGA and HGA cases in 2021. While Campeau et al. (2022) highlight the need to include GA as a Canadian human reportable disease to support early identification of outbreaks, we aim to push this reflection further. We think that this article illustrates the importance of integrating environmental data (i.e., questing ticks) in TBD surveillance systems to provide early signals of TBD emergence and inform timely public health response, and provide evidence on the added value of the One Health approach to TBD surveillance.

## Contributions to knowledge

What does this study add to existing knowledge?

- Analyses of questing *I*. *scapularis* ticks presented in this article showed an increase by 5.7% in the prevalence of *Anaplasma phagocytophilum*, one year before the occurrence of an outbreak of animal and human granulocytic anaplasmosis in southern Québec.
- More than 60% of positive ticks were infected by the strain pathogenic for humans, dogs, and horses.

What are the key implications for public health interventions, practice, or policy?

- The study provides evidence on the added value of the One Health approach to TBD surveillance. It suggests that the early detection of TBD emergence requires (i) implementing and maintaining active field surveillance in regions at high risk for TBD and (ii) targeting, within these regions, areas with endemic *I*. *scapularis* tick populations and investing significant sampling efforts.

## Supplementary Information

ESM 1 (DOCX 28 kb)

## Data Availability

Data are available on reasonable request.
