# Supplementary material for: The added value of One Health surveillance: data from questing ticks can provide an early signal for anaplasmosis outbreaks in animals and humans
Source: Can J Public Health. 2022 Dec 5;114(2):317–24. doi: 10.17269/s41997-022-00723-8 (PMC10036682; doi:10.17269/s41997-022-00723-8)
Supplement: Supplementary file 1 — (DOCX 28 kb) [file 41997_2022_723_MOESM1_ESM.docx]

**Treatment of small mammals**

The analysis described in the article were done on some ticks, nymphs and adults, collected from sites where small mammals were exposed to an acaricidal treatment. The objective of the treatment is to reduce the density of infected ticks by the causative bacterial agent of Lyme disease. However, it has the potential to affect all pathogens maintained and amplified in the environment by small mammals such as mice of the genus *Peromyscus*. This treatment was deployed on 10 sites out of 11 in 2019, on 10 out of 15 in 2020 and on 9 out of 14 in 2021. All landscape located within sites with treatment application are not exposed to the treatment. One third of site landscapes with treatment application have acaricidal baits deployed. The remaining 2/3 are landscape exploring the spatial extent of the treatment. In this file, we present the number of ticks analysed, the prevalence of Ap-positive ticks and the proportion of Ap strain among Ap-infected specimens stratified by treatment stratus (Table S1). To simplify presentation of the data, ticks were classified according to their distance from a landscape where acaricidal baits were deployed. Ticks located at more than 175 meters were classified as *no treatment* (it includes ticks from sites where no bait were deployed), ticks between 175 m and 25 m were classified as *nearby treatment* and ticks within a range of 25 m of treated landscape were classified as *treatment*.

**Table S1. Data from ticks included in the analysis presented in the article stratified according to their distance from landscape with treatment**

| **Exposition** | **No. tested** | **Ap^a^** | **Prevalence (%) [CI95]** | **Ap-ha^a^** | **Proportion (%) [CI95]** |
| --- | --- | --- | --- | --- | --- |
| Treatment [0 - 25[ | 269 | 11 | 4.1 [2.1 - 7.1] | 6 [10]^b^ | 60.0 [26.2 - 87.8] |
| Nearby treatment ]25 - 175[ | 227 | 16 | 7.0 [4.1 - 11.2] | 10 [15] | 66.7 [38.4 - 88.2] |
| No treatment [175 - | 372 | 25 | 6.7 [4.4 - 9.8] | 6 [9] | 66.7 [29.9 - 92.5] |

^a^Number of Ap or Ap-ha positive ticks

^b^Number of Ap-positive samples for which the strain was successfully identified
